# Comparative Evaluation of Two Venous Sampling Techniques for the Assessment of Pancreatic Insulin and Zinc Release upon Glucose Challenge

**DOI:** 10.1155/2015/789359

**Published:** 2015-07-27

**Authors:** Anil Kumar Pillai, William Silvers, Preston Christensen, Matthew Riegel, Beverley Adams-Huet, Ildiko Lingvay, Xiankai Sun, Orhan K. Öz

**Affiliations:** ^1^Department of Radiology, University of Texas Southwestern Medical Center, Dallas, TX 75390, USA; ^2^Animal Resources Center, University of Texas Southwestern Medical Center, Dallas, TX 75390, USA; ^3^Departments of Internal Medicine and Clinical Sciences, University of Texas Southwestern Medical Center, Dallas, TX 75390, USA; ^4^Advanced Imaging Research Center, University of Texas Southwestern Medical Center, Dallas, TX 75390, USA

## Abstract

Advances in noninvasive imaging modalities have provided opportunities to study *β* cell function through imaging zinc release from insulin secreting *β* cells. Understanding the temporal secretory pattern of insulin and zinc corelease after a glucose challenge is essential for proper timing of administration of zinc sensing probes. Portal venous sampling is an essential part of pharmacological and nutritional studies in animal models. The purpose of this study was to compare two different percutaneous image-guided techniques: transhepatic ultrasound guided portal vein access and transsplenic fluoroscopy guided splenic vein access for ease of access, safety, and evaluation of temporal kinetics of insulin and zinc release into the venous effluent from the pancreas. Both techniques were safe, reproducible, and easy to perform. The mean time required to obtain desired catheter position for venous sampling was 15 minutes shorter using the transsplenic technique. A clear biphasic insulin release profile was observed in both techniques. Statistically higher insulin concentration but similar zinc release after a glucose challenge was observed from splenic vein samples, as compared to the ones from the portal vein. To our knowledge, this is the first report of percutaneous methods to assess zinc release kinetics from the porcine pancreas.

## 1. Introduction

In 2013, the prevalence of diabetes mellitus was 382 million people worldwide and is expected to rise to 592 million by 2035, an increase of 55% [1]. Approximately 37 million persons in the United States have diabetes, of which type 2 accounts for the vast majority (90%) of cases. According to the Centers for Disease Control and Prevention, diabetes mellitus is the 7th leading cause of mortality and contributes to 38% of deaths before the age of 60 in the United States [2]. Early detection and lifestyle changes can have a positive impact in preventing complications of diabetes.

Advances in noninvasive imaging modalities have provided opportunities to study the pancreatic *β* cell function through imaging zinc release from *β* cells, as it is coreleased with insulin [3, 4]. The highest concentration of zinc in the body has been detected in the pancreatic *β* cells within the Islets of Langerhans, which is crucial for the synthesis, secretion, and signaling of insulin [5–7]. Alterations to zinc transporters on *β* cells have been linked to altered insulin synthesis, secretion, and increased risk of diabetes [8–10]. The insulin response to a rapid intravenous glucose challenge has been reported to be biphasic, where the first phase results from the release of preformed insulin granules [11, 12]. The subsequent phase, which is more sustained, is due to mobilization of a stored granule pool. During insulin secretion, zinc is coreleased into the extracellular islet space [13]. The temporal dynamics of zinc release from *β* cells have been studied at the cellular level using isolated islets [1], but little is known about the dynamics of zinc release in vivo. Imaging of zinc release as a surrogate marker for insulin secretion is the basis for developing imaging probes to noninvasively monitor *β* cell function [3]. Understanding the temporal secretory pattern of zinc and insulin corelease after a glucose challenge is essential for proper timing of probe administration. Continuous peripancreatic venous sampling after glucose challenge provides an accurate temporal profile of zinc release, whereby the biphasic response could be assessed.

Animal models serve as an important research tool for nutritional physiological studies, in which swine are commonly used owing to the numerous anatomical and physiological similarities between porcine and human [14]. Traditionally, portal vein sampling in swine involved a surgical procedure including an abdominal incision and placement of a vascular access port for repeated sampling [15, 16]. Herein, we describe two different percutaneous techniques to perform blood sampling from the portal and splenic veins and compare the temporal sequence of insulin and zinc release after a glucose challenge in each technique.

## 2. Materials and Methods

All experimental procedures involving animals were approved by the UT southwestern institutional animal care and use committee (IACUC). The procedure was performed on six adult Ossabaw pigs weighing between 80 and 260 pounds under general anesthesia [17].

### 2.1. CT Angiography

CT angiography of the first pig was done to evaluate the normal portal anatomy for planning the portal venous access. The images were acquired on a Siemens Biograph PET/CT scanner equipped with a 64-slice CT scanner (Siemens Healthcare USA, Inc., Malvern, PA). The right femoral vein was accessed under ultrasound guidance. Over a guidewire, a 5-French straight flush catheter was advanced and the tip positioned in the mid caudal vena cava. Catheter position was confirmed on the scout scans. A contrast agent (Omnipaque 350, GE Health care, Princeton, NJ) was injected at 3 mL/sec for a total volume of 154 mL (2 mL/kg). Image acquisition was performed at 18 seconds for the arterial phase and 29 seconds for the portal venous phase [18]. Images were reconstructed using a Siemens clinical workstation in the TrueD CT software environment.

### 2.2. Venous Sampling Techniques

The portal vein sampling was done in a dedicated fluoroscopy animal suite (Infinix AX, Toshiba Inc., Tokyo, Japan). Ultrasound guided saphenous venous access for blood draws and glucose infusion was obtained prior to the procedure. Two different techniques were used for portal venous sampling as described below.

#### 2.2.1. Technique 1: Transhepatic Ultrasound Guided Portal Vein Access

Each of the three pigs sampled by this technique was positioned on the fluoroscopic table on the left lateral decubitus position. The right upper abdomen was shaved and cleaned. Preprocedure ultrasound scan using a 3.5 mHz curvilinear transducer (Siemens Elegra, Erlangen, Germany) was performed to identify a suitable portal vein branch (right, left, or main portal vein). Using an intercostal approach, a 21-gauge needle was advanced under real time visualization into the targeted portal vein. Two methods were used to visualize the needle while performing the ultrasound guided access: in-plane (long axis) and out-of-plane (short axis). The former follows the tip of the needle, which was seen as a bright spot. As the tip was being advanced, the probe was angulated in the direction of the needle tip in small increments until the tip was visualized within the target. The latter visualizes the entire needle. The needle was kept in the same plane as the ultrasound beam. By adjusting the needle to be in-plane with the ultrasound beam while keeping the target in the field of view, the needle was advanced into the target. After fluoroscopic confirmation of the access by injecting 5–10 mL of Omnipaque 350, a 0.018-inch wire was passed into a branch of the right or left portal vein, over which a 5-French (F) triaxial introducer (Neff set, Cook, Bloomington, IN) was advanced to establish the access. Procedure time was defined as the duration from the initial needle stick as recorded by the anesthesia team in their procedure log to the time stamp on the fluoroscopic image confirming the venous access. Through the introducer sheath, a 4 F angled catheter was advanced for blood sampling.

#### 2.2.2. Technique 2: Transsplenic Fluoroscopy Guided Splenic Vein Access

Each of the three pigs sampled by this technique was positioned on the fluoroscopic table in the left lateral decubitus position. The left upper abdomen was shaved and cleaned. Preprocedure ultrasound scan using a 3.5 mHz curvilinear transducer (Siemens Elegra, Erlangen, Germany) was performed to identify the spleen. Using an intercostal approach, a 21-gauge needle was advanced under real time visualization into the splenic parenchyma. Omnipaque 350 (5–10 mL) was gently injected into the splenic parenchyma under real time fluoroscopy until the splenic vein was opacified (Figure 3(a)). A second 21-gauge needle was advanced into the splenic vein under fluoroscopic guidance using a triangulation technique [19]. The C arm was moved back and forth between 2 orthogonal positions: 1 parallel and 1 oblique to the line of puncture. This orients the operator if the needle is off plane in the mediolateral (parallel) or craniocaudal (oblique) plane. Adjustments can then be made in several small steps until the needle hits the target (Figure 3(b)). Once the needle was visualized within the targeted splenic vein, 5–10 mL of Omnipaque 350 was injected and a venogram performed to confirm access. A 0.018-inch wire was passed into the splenic vein, over which a 5 F triaxial introducer (Neff set, Cook, Bloomington, IN) was advanced to maintain the access. Procedure time was defined as the duration from the initial needle stick as recorded by the anesthesia team in their procedure log to the time stamp on the fluoroscopic image confirming the venous access. Sampling was done through this sheath.

### 2.3. Measurement of Zinc and Insulin Concentration

After venous access was established, 2 mL of blood was obtained from the portal/splenic vein for baseline measurements. A dextrose bolus (0.3 g/kg) was administered within 20 seconds into the saphenous vein. The first sample was obtained 2 min after the dextrose injection. Sampling was subsequently performed every 2 min for a total of 30 samples. Plasma samples were prepared from the blood samples for the measurement of zinc and insulin concentrations. Plasma zinc concentration was measured using an inductively coupled plasma mass spectrometer (ICP-MS, Agilent 7700, Santa Clara, CA, USA). Zinc samples were prepared by diluting 100 *µ*L of plasma into 900 *µ*L of 10% hydrochloric acid (HCL) (trace metal, 3.6 ppb Zn) in nanopure water (Millipore Gradient Milli-Q water system, Billerica, MA). Samples were vortexed to mix thoroughly and centrifuged at 14,000 rpm for 10 min to remove lipid and protein pellets. The results were determined using a calibration curve generated from a commercially available zinc standard (Inorganic Ventures, Christiansburg, VA USA). Insulin concentration in the plasma samples was measured using commercially available porcine insulin ELISA kits (Alpco).

### 2.4. Statistics

The variables evaluated in the two groups were ease of access as measured by the time to establish access (catheter positioned in desired location) and concentration of insulin and zinc in the plasma samples. The ease of access was determined using the average time to establish access after the start of the procedure. Zinc and insulin concentrations were plotted versus time after the dextrose challenge and the curves were fitted with spline regression using cubic splines with six knots (SAS version 9.4, SAS Institute, Cary NC, USA). The mean concentrations obtained using the two techniques were compared with a mixed effect linear model with vein and time main effects and modeling the pig as a random effect; a *p* value of <0.05 was considered statistically significant. Results are summarized as mean and standard deviation unless otherwise indicated.

## 3. Results

### 3.1. Pancreatic Venous Drainage Sampling

It is not feasible to assess each islet that is surrounded by microcirculation in the whole pancreas. However, local whole organ venous drainage can be percutaneously sampled. In this way, the release kinetics of insulin and zinc upon glucose stimulation can be assessed with minimal influence of dilution by systemic blood. By such a sampling approach, we anticipated to determine the temporal window that allows an imaging probe for noninvasive assessment of *β* cell function through zinc release upon glucose stimulation.

**Figure 1:**
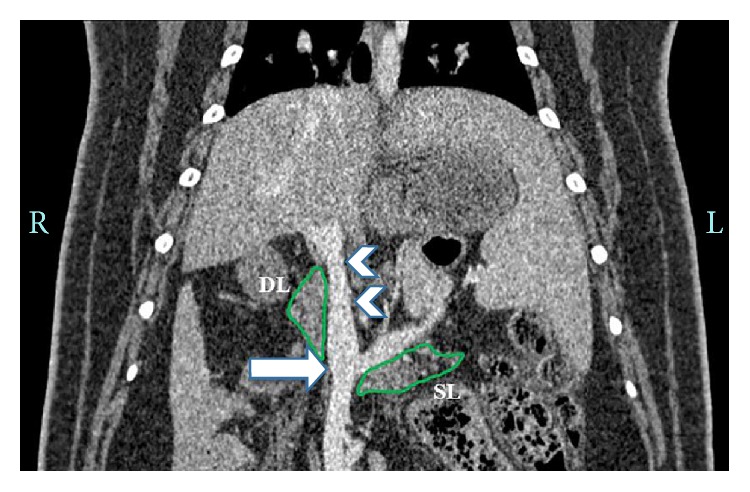
Preinterventional planning CT angiography. Coronal CT angiogram demonstrating the normal vascular anatomy of the portal venous system. Note the splenoportal confluence (arrow) and the long extra-hepatic course of the portal vein (arrowheads) in relation to the pancreatic duodenal lobe (DL) and the splenic lobe (SL). Both pancreas lobes are outlined in green.

Prior to interventional radiology procedures, CT angiography was undertaken to obtain an idea of venous anatomy in the upper abdomen of the pig.  Figure 1 is a representative illustration of the portal venous anatomy vasculature in the upper abdomen of an Ossabaw pig along with its spatial relation to the pancreatic splenic lobe (SL) and duodenal lobe (DL). The splenic vein is partially bounded by the SL of the pancreas and unites with the superior mesenteric vein to form the portal vein, to which the splenic vein drains [20]. The DL of the pancreas rests against the base of the portal vein, which has a 2-3 cm extra-hepatic course and divides at the hepatic hilum into the right and left portal veins.

**Figure 2:**
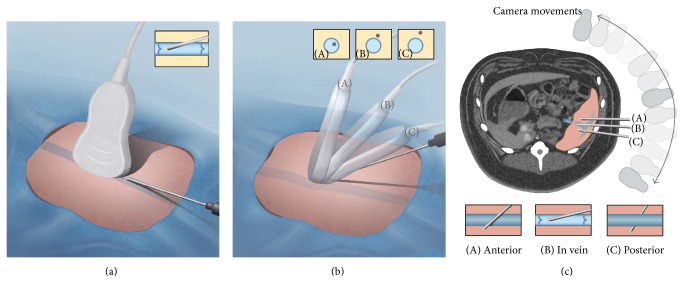
Illustration of ultrasound guided venous access. (a) Illustration demonstrating the in-plane technique for the ultrasound guided access. The inset shows a schematic of the needle within the vein in long axis. (b) Illustration demonstrating out-of-plane technique for ultrasound guided access. The inset shows a schematic of the blood vessel in cross section with the dot being the needle position in the respective transducer locations. (c) Overlay illustration on an axial CT image demonstrating the relationship of the needle to the venous structure, with different positions of the fluoroscope C arm. The pink shading over the CT image denotes the spleen.

**Figure 3:**
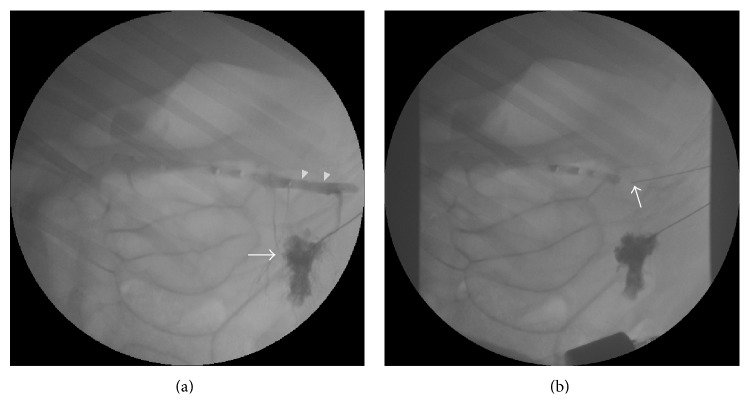
Transsplenic splenoportal venous sampling technique. (a) Digital fluoroscopic image demonstrating parenchymal blush with reflux of contrast through the splenic parenchyma opacifying the splenic vein (white arrowheads). (b) Digital fluoroscopic image demonstrating the passage of the second needle targeting the splenic vein.

The transhepatic portal vein access was successfully performed (Figure 2). The main portal vein was accessed in 2 pigs and the right portal vein was accessed in 1 pig. Samples were obtained from the distal portal vein. The transsplenic access to the splenic vein was equally successful (Figure 3). The 2-needle technique was quicker and easier to perform. The mean (SD) time taken to access the splenic and portal veins was 34 (14) and 50 (15) min, respectively. The average number of needle passes to obtain a satisfactory access into the splenic vein after opacification was 2. No procedural or postprocedural complications were observed in either sampling group and all pigs recovered equally well within a similar amount of time.

### 3.2. Comparative Kinetics of Insulin and Zinc Release Obtained by the Two Sampling Techniques

**Figure 4:**
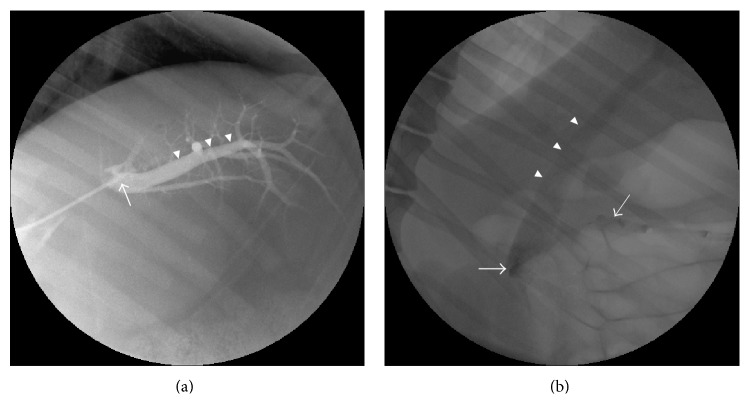
Angiographic demonstration of venous sampling sites. (a) Digital fluoroscopic image demonstrating the catheter tip (white arrow) in position for obtaining samples from the distal left portal vein (white arrowheads). (b) Digital fluoroscopic image demonstrating the splenoportal confluence (white thick arrow). Note the opacified splenic vein (white thin arrow) and the long extra-hepatic course of the portal vein (white arrowheads).

**Figure 5:**
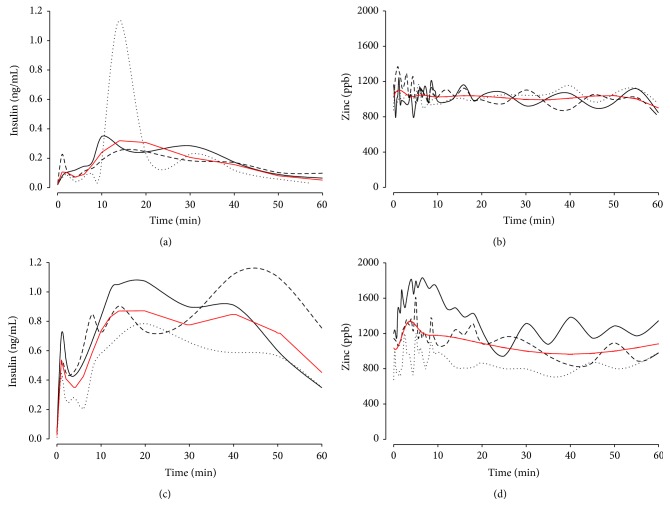
Temporal profile of insulin and zinc concentrations in the portal or splenic blood pool after injection of a dextrose bolus. Three pigs per sampling method are represented by the black dotted, dashed, and solid lines. The red curves shown are spline fits. Top row insulin (a) and zinc (b) release profiles from the transhepatic portal venous sampling technique. Bottom row insulin (c) and zinc (d) release profiles from the transhepatic splenic venous sampling technique. Note the biphasic insulin response with an associated clear rise of zinc concentration after the first peak at approximately 5 minutes.

In the portal sampling technique, all the samples were obtained from the distal branches of the left portal vein (Figure 4(a)). Average baseline plasma zinc and insulin concentration were 1019 ± 150 ppb and 0.024 ± 0.006 *µ*g/mL, respectively. A biphasic pattern in insulin release after the glucose challenge was noted as shown in the spline fitted results (red line tracings) (Figure 5). The first peak occurred at 2 min and the second was predicted to occur at 16 min. The mean predicted concentrations were 0.10 and 0.32 *µ*g/mL, respectively. In the zinc release profile, there was more scatter in the data and only a single well defined peak was identified at around 1.3 min to a mean value of 1103 ppb.

In the splenic vein sampling technique, all samples were obtained from the splenoportal junction (Figure 4(b)). Average baseline plasma zinc and insulin concentration were 1001 ± 298 ppb and 0.032 ± 0.023 *µ*g/mL, respectively. Samples from the splenic vein had higher insulin (*p* < 0.0001) but similar zinc concentration (*p* = 0.49) compared to the portal vein. The anticipated biphasic insulin release profile was again observed (Figure 5). The insulin concentration peaked at 2 min after the glucose challenge to a predicted value of 0.56 ± 0.14 *µ*g/mL, an approximately 18-fold increase of the mean. The zinc release profile again showed a single clear peak that followed the first insulin peak and was at 4 min with a mean value of ~1338 ppb. The confidence interval was wider than for the portal sampling technique.

## 4. Discussion

Portal venous sampling is an essential part of pharmacological and nutritional studies. In the work presented here, portal venous sampling was utilized to find the temporal relationship of zinc release from the pancreatic *β* cells upon glucose.

Portal venous access in swine was first described in 1969 by Lydtin et al. [21]. They implanted a catheter in the portal vein for repeated sampling; however, the intent was not to define the kinetics of insulin or zinc release. Over the years, many others have described techniques for portal venous sampling using various permanent implantation devices. These procedures involved open surgical techniques with associated complications of postoperative sepsis and thrombosis [22]. As demonstrated in this study, percutaneous splanchnic venous sampling is straightforward, is easily reproducible in different pigs, and can be performed efficiently and safely. While both transhepatic and transsplenic image-guided techniques were reproducible and safe, sampling from the splenic vein via transsplenic access took less time and was technically easier to perform.

It is known that insulin release from the pancreatic *β* cell after glucose challenge is biphasic. The first phase of the insulin response is the result of release of preformed granules. This phase is almost invariably reduced in patients with impaired glucose tolerance test or in early stages of type 2 diabetes before overt symptoms of the disease manifest [23]. In our study, we observed a biphasic pattern of insulin release using either the transhepatic technique with portal venous sampling or the transsplenic technique with splenic sampling. Additionally, the splenic vein samples had higher mean concentration peak values for both insulin and zinc. This observation possibly resulted from dilution by the mixing with blood from the superior mesenteric vein. Sampling without mesenteric venous contamination can also be achieved from the portal venous access by placing a catheter under fluoroscopic guidance into the splenoportal confluence from the portal access site. The fact that the distal portal vein radicles are smaller than the splenoportal confluence might also contribute to the sampling errors and also explain the higher concentration of insulin and zinc in the splenic vein cohort. The temporal secretory correlation between insulin and zinc was inconsistent in both cohorts. The correlation was numerically, but not statistically, higher in the splenic vein cohort. The explanation of the inconsistency is presently unclear but may reflect a physiological range in insulin and zinc correlations in pigs. Nevertheless, we achieved one of our aims which was to define a time interval for administration of zinc targeting radiotracer imaging after a glucose challenge. Based on our results, an optimal window for injection of a tracer targeting zinc release would be in the 2–5 min time frame.

Our study has some limitations. Firstly, initially there were animal welfare concerns that caused us to perform the two techniques on different pigs. Based on our experience in this study, a pig could undergo venous sampling by both techniques. An added benefit of this study design could be mitigation of variation caused by physiological differences in animals. Secondly, the sample sizes in each group were small.

In summary, our study assessed the temporal pattern of insulin and zinc release after glucose challenge using 2 different techniques for venous sampling. These techniques use small catheters, which are not traumatic and can be removed after the sampling is performed without any complications. It should be possible to safely perform repeated access in the same animal. Based on the data from two cohorts of Ossabaw minipigs, we conclude that the splenic vein access for sampling is technically easier and superior to transhepatic portal vein access for studying *β* cell function after glucose challenge. To our knowledge, neither the biphasic insulin release profile nor the temporal profile of zinc release in response to a glucose challenge has been reported in swine as demonstrated here. The splenoportal venous sampling via the transsplenic access technique presented herein may find applications in testing the efficacy of therapeutics aimed at improving *β* cell function.

## Supplementary Material

Video File 1 Transhepatic portal venous access, ultrasound guided long axis method: The probe and the needle are placed side by side. The portal vein (dark echo void rounded structure) is almost 15 cm deep (the distance markers are on the left side of the screen, long markers every 5 cm and short markers every 1 cm from the skin). The needle is seen traversing the liver parenchyma towards the portal vein on the right side of the screen as an echogenic moving spot. Note, the beam follows a sector pattern and the needle is kept within the beam. During respiratory moments the needle will move in and out of plane. The operator adjusts the needle by minor cranio-caudal and medio-lateral deflections to keep the needle in plane while advancing the tip towards the portal vein. Contrast injection confirms portal vein access.
Video File 2 Transplenic splenic vein access: First needle is passed into the splenic parenchyma. Contrast injected through this needle to opacify the splenic parenchyma with reflux into the splenic vein. Targeting the opacified splenic vein, a second needle is advanced in this plane. The needle is observed on real time fluoroscopy as it passed into the target. The needle tip is then confirmed to be within the targeted splenic vein in all projections by rotating the C arm while observing it's relation to the opacified splenic vein on live fluoroscopy. If the tip is too deep or too shallow, appropriate needle adjustments are performed. Contrast injection confirms splenic vein access.
